# The effects of intrinsic foot muscle strengthening interventions for adults over age 65: a randomized controlled trial protocol

**DOI:** 10.3389/fragi.2025.1622232

**Published:** 2025-10-15

**Authors:** Erin Futrell, Yvonne Golightly, Yara Haddad, Andrea Carmichael, David Taylor

**Affiliations:** ^1^ Foot Intrinsic Testing and Training Lab, Springfield College, Department of Physical Therapy, Springfield, MA, United States; ^2^ University of Nebraska Medical Center, College of Allied Health Professions, Omaha, NE, United States; ^3^ Division of Injury Prevention, National Center for Injury Prevention and Control, Centers for Disease Control and Prevention, Atlanta, GA, United States; ^4^ Mercer University, Department of Physical Therapy, Atlanta, GA, United States

**Keywords:** older adult, falls, foot, footwear, intervention

## Abstract

Unintentional falls are the leading cause of injury in adults ≥65 years. While causes of falls are multifactorial, weakness or disuse of the intrinsic foot muscles (IFM) can contribute. The purpose of this randomized controlled trial, using an effectiveness-implementation hybrid design, is to analyze the effects of two IFM strengthening interventions (minimal footwear use or strengthening exercises) on IFM size, proprioception, foot structure, and fall risk in older adults. Adults ages ≥65 years, with fall risk, who can ambulate household distances (with an assistive device as needed), will be invited to participate. Individuals with poor foot sensation, vestibular disorders, lower extremity amputation, lower extremity or lumbar spine injury or surgery in the previous 6 months, impaired cognitive ability to follow verbal and written instructions, and those who have participated in a fall prevention program in the past 6 months will be excluded. Participants will be randomly allocated into three groups: prescribed minimal footwear use, IFM strengthening exercises, or control. Participants will be encouraged to perform their intervention 5 days per week for 16 weeks, and then at least 2 days per week from 17 weeks to 1 year. Participants will be asked to record intervention performance, daily step count, and falls in provided diaries. At baseline, 8 and 16 weeks, and 1 year, participants will undergo measurements of IFM cross-sectional area (CSA, cm^2^) using ultrasound imaging, proprioception, foot structure (navicular drop and hallux valgus angle) and fall risk. Semi-structured interviews will be conducted and recorded to gain participant impressions of the interventions, self-reported effects of the interventions, and impressions of study activities to inform future research and clinical implementation. A three-*group* x four *time point* repeated measures analysis of covariance will be used to assess changes in measurements. Implementation data will be analyzed using both quantitative and qualitative methods. This will be the first study among older adults to assess the effects of IFM strengthening interventions on long-term fall risk and proprioception, and to use ultrasound imaging to assess IFM size changes. These interventions are simple, safe, and affordable and may have a major impact on functional mobility and reduction of falls for older adults.

## Introduction

Unintentional falls are a leading cause of death, injury, and reduced function in adults aged 65 years and older (older adults). (CDC WISQARS) Falls among older adults are multifactorial, but include age-related changes to the musculoskeletal and neuromuscular systems that impair strength, balance, and stability (Laurence, 2021). Foot alignment and muscle function play a key role in stability, balance, and mobility (Spink et al., 2011).

The 22 intrinsic foot muscles (IFM) provide stability to the larger extrinsic foot muscles to produce gross movements such as walking (McKeon et al., 2015). The plantar IFM help the foot adapt to surfaces, stabilize the foot’s arches, and provide somatosensory input for motor planning and adaptations during balancing and walking (McKeon et al., 2015; Jastifer, 2023). Proprioceptors, sensors within various body tissues, including the IFM, provide sensations of movement, position, force, effort, and balance (Proske and Gandevia, 2012). Weakness or disuse of the plantar IFM can lead to abnormal motion or deformities of the foot and toes, arch instability, foot pain, and potentially, falls (Spink et al., 2011; Glasoe, 2016; Menz et al., 2006; Mickle et al., 2009).

In healthy older adults, IFM are smaller and weaker than in younger adults.^10 11^ Toe deformities and/or IFM weakness in older adults are determinants of balance and functional ability, and more importantly, independent predictors of falls (Spink et al., 2011; Menz et al., 2006; Mickle et al., 2009). An extensive 2024 review by the US Preventive Services Task Force found that exercise interventions reduce falls by a moderate amount (Guirguis-Blake et al., 2024), but of the 70 exercise references, only two mentioned the use of “thin” footwear (which may naturally recruit IFM), and none mentioned foot strengthening exercises (Guirguis-Blake et al., 2024). A recent systematic review reported little evidence exists on targeted IFM strengthening interventions in older adults (Futrell et al., 2022). Given the paucity of literature exploring the IFM’s role in fall prevention, further investigation of this underexplored muscle group is warranted. Should findings support IFM strengthening, it could easily be added to existing exercise-based fall prevention programs (Futrell et al., 2022).

Simple, safe, and inexpensive interventions that address IFM weakness have been implemented successfully in small samples of younger and older adults (Futrell et al., 2022). These include isolated IFM strengthening exercises, or the use of minimal footwear during daily tasks. Both interventions are speculated to not only increase strength of the IFM, but also enhance proprioception. Proprioception declines with age and contributes to falls, but similar to muscle strength, it can be improved with tissue-specific interventions (Proske and Gandevia, 2012).

**FIGURE 1:**
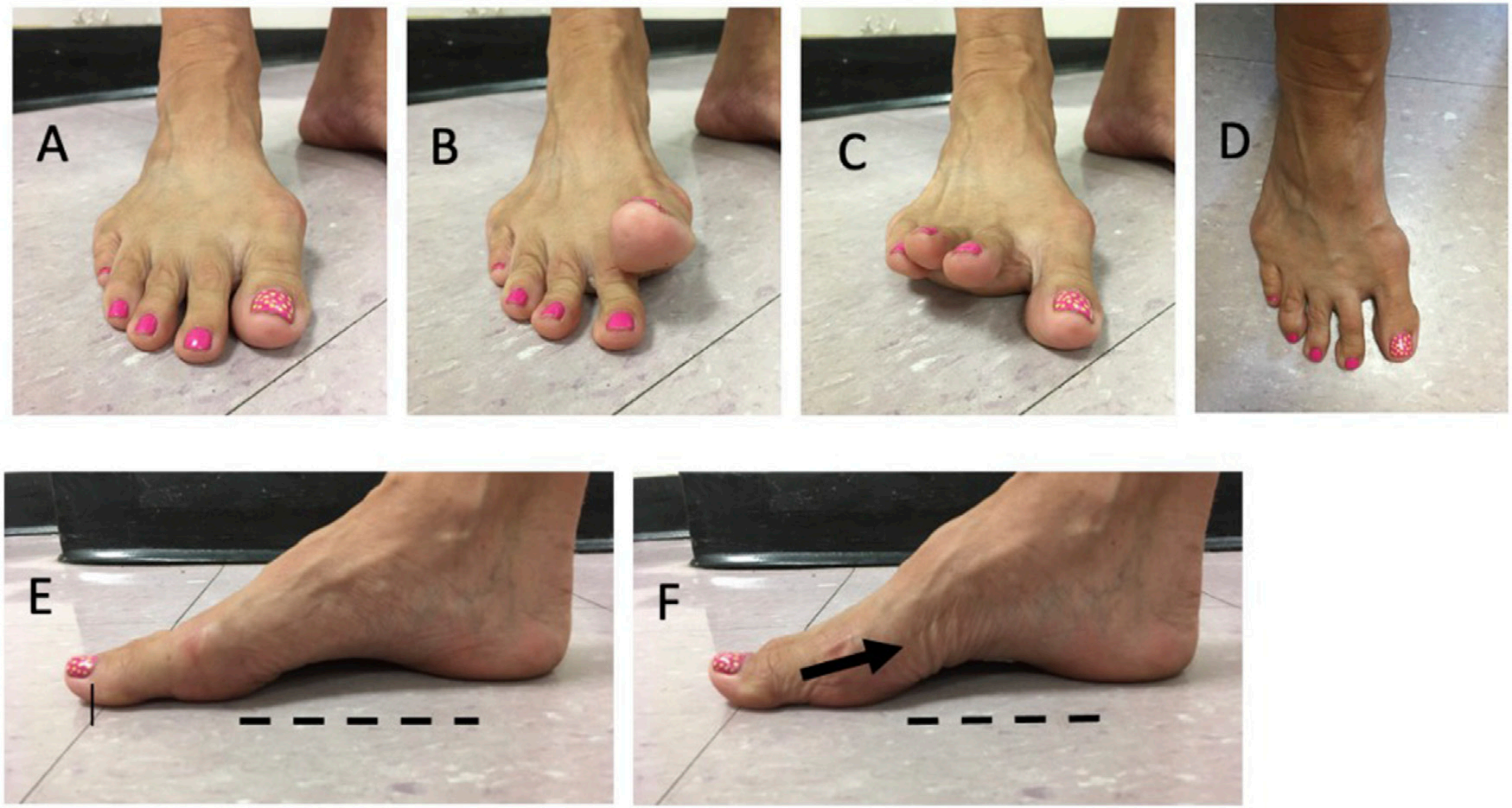
Examples of intrinsic foot muscle (IFM) strengthening exercises: **(A)** foot at rest; **(B)** great toe lift; **(C)** small toes lift; **(D)** toe spread; **(E)** foot at rest; **(F)** short foot exercise or “doming” – note the skin folds as the muscles contract and creates greater arch height.

Minimal footwear has little to no cushioning, a wide toe box, and simulates barefoot conditions while protecting the sole. This footwear allows maximal motion and flexibility with more sensory input to the foot, which may naturally engage and recruit the IFM during daily activities. IFM exercises include the short foot exercise and a series of toe movements, sometimes called “toe yoga” (Figure 1) (McKeon et al., 2015; Glasoe, 2016; Kim et al., 2013; Gooding et al., 2016) Isolating the IFM with specific exercises may improve foot and toe alignment and influence motor control, which may not be achieved with combined extrinsic-intrinsic activities (such as towel crunches) because they require less control (Fraser and Hertel, 2019a).

In two studies by Ridge et al. of young healthy adults, they found that minimal footwear use with daily activities increased IFM size and strength as much as IFM strengthening exercises (Ridge et al., 2018), and that progressive time spent walking in minimal footwear increased IFM size and strength (Ridge et al., 2019). While the effects in older adults are not yet known, a small study of 4 months of minimal footwear use in older adults showed improvements in lower extremity pain, foot sensation, and overall function (Franklin et al., 2017a; Trombini-Souza et al., 2015). Researchers speculated the minimal footwear may enhance neuromuscular stimulation or proprioception in the feet, leading to improvements in gait and balance—key protective factors for falls (Fraser and Hertel, 2019b; Arnold and Menz, 2023).

Limited research on IFM strengthening exercises for older adults is promising, demonstrating increased IFM strength with supervised training (Futre et al., 2022; Mickle et al., 2016b). Increased large muscle group cross-sectional area (an indication of hypertrophy and increased strength) occurred in as little as 6–9 weeks of strength training in older adults (Mayer et al., 2011). The same may be possible for the IFM in an older population, although it may take longer in deconditioned or frail individuals (Borst, 2004). It is unknown if IFM strength increases, with minimal footwear or specific exercises, result in reduction of falls, fall risk, or improved mobility.

The objective of this study is to assess the effects of two IFM strengthening interventions in older adults compared to a control intervention, focusing on the effects on IFM cross-sectional area (CSA), proprioception, foot structure, and fall risk. Results may inform fall prevention interventions for older adults. This study is registered at ClinicalTrials.gov (NCT05829369). The study received Institutional Review Board approval by the lead investigator’s institution (Springfield College, Springfield, MA), approval #1492223. The protocol for this project follows the Consolidated Standards of Report Trials (CONSORT) checklist.

## Methods

### Study design

This is a randomized controlled trial with an effectiveness-implementation hybrid design Type I (Curr et al., 2012). The Type I hybrid design aims to “test the effects of a clinical intervention on relevant outcomes while observing and gathering information on implementation” (Curr et al., 2012). Highly controlled intervention effectiveness studies typically hold good internal validity, but often lack external validity and generalizability. Testing an intervention while also researching its implementation may help to expedite gains in knowledge and address gaps between research and clinical practice, as well as inform decision makers in these fields.

### Sample size

An *a priori* power analysis (α = 0.05, power = 80%) was performed for the primary measure of interest: fall risk based on Mini-Balance Evaluation Systems Test (Mini-BESTest) scores. A study by Conradsson, et al. that measured fall risk with the Mini-BESTest following a fall prevention intervention, found a 3-point change in Mini-BESTest scores in the intervention group and a large effect size (Cohen’s d = 0.82) (Conradsson et al., 2015). In addition, Godi et al. performed a reliability, validity, and responsiveness study of the Mini-BESTest and determined the minimal detectable change (MDC) at the 95% confidence interval was 3.5 points (Godi et al., 2013). Based on these previous findings, the power analysis indicated *at least 22 participants per group are needed*. To account for 30% participant attrition, 30 participants per group will be targeted (3 groups, total of 90 participants).

### Participants

Recruitment of community-dwelling older adult participants will occur through written English and Spanish language materials (flyers, posters, e-mail, and newspaper advertisement) and in-person group information sessions. Recruitment will occur in several places in the greater metropolitan area of Springfield, MA in an effort to have a diverse population of older adults in terms of sex, race, ethnicity, socioeconomic background, and education level. These places will include a local assisted living facility, several local senior centers, and healthcare facilities affiliated with Baystate Medical Center, Springfield, MA. Recruitment will be ongoing until the specified sample size is met (n = 90).

Before conducting any research activities, participants will be informed of the details of the study, permitted to ask questions, and sign an informed consent document. Participation in the study is expected to last for 12 months from the time of baseline measures.

Inclusion criteria are:

Adults aged ≥65 years who can ambulate household distances (16 m) with or without an assistive device

Have fall risk based on either:

a. a “yes” to any of the Three Key Questions (Burns et al., 2022) [1) Do you feel unsteady when standing or walking? 2) Do you worry about falling? 3) Have you fallen in the past year?] or
b. Have fall risk based on a Timed Up and Go score ≥12 s (CDC, 2023).

Participants will be excluded from participation if they have any of the following:

• Poor foot sensation based on Semmes-Weinstein monofilament testing (Singh et al., 2005) (at least 5 of 9 sites on plantar foot have intact light touch sensation with 5.07/10 g filament).
• Lower extremity amputations.
• Lumbar spine or lower extremity injury or surgery in the past 6 months.
• Impaired cognitive ability to follow verbal or written instructions based on the Mini Mental State Exam (score <24) (Tombaugh and McIntyre, 1992).
• Vestibular disorders based on self-report or the Vestibular Screening Tool (score ≥4) (Stewart et al., 2015).
• Participation in a fall prevention program in the previous 6 months.

### Setting

Screening, enrollment, and measurement sessions will occur in a physical therapy research laboratory (The Foot Intrinsic Testing and Training Lab) on the campus of Springfield College, Springfield, MA. In addition, a local assisted living center in Springfield, MA has agreed to allow all study activities to occur within their facility for interested residents and non-residents. Participants will have their choice of the most convenient location.

**FIGURE 2:**
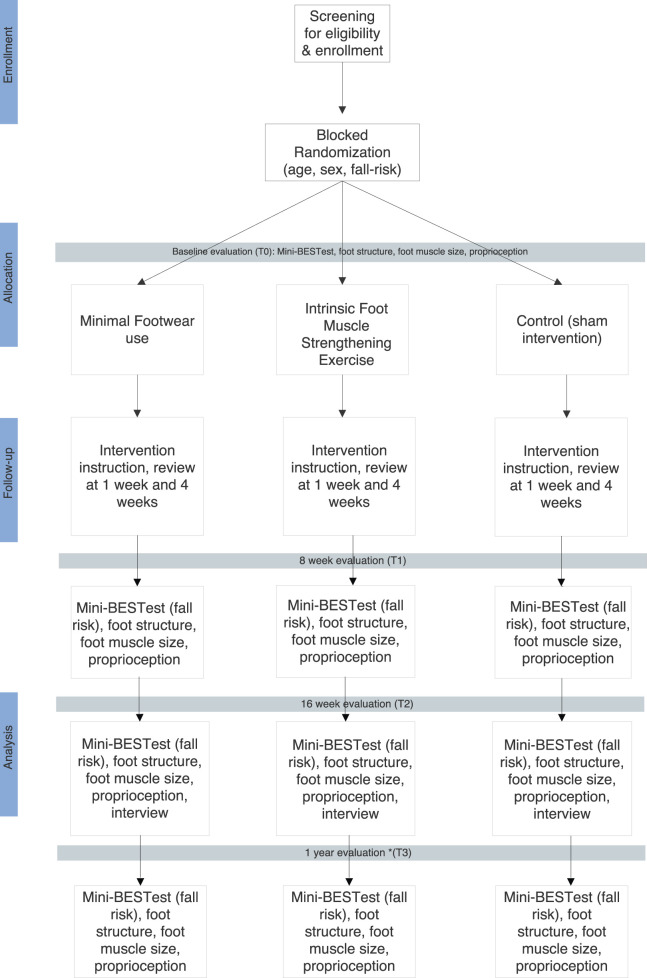
Older adult foot strengthening to prevent falls study protocol, 2023–2025.

Figure 2 presents an overview of the study.

#### Randomization

Following screening and enrollment, participants will be allocated to one of three groups using block randomization based on sex (male/female), age (65–84 or ≥85 years) and pre-intervention fall risk based on Mini-BESTest scores (mild risk 28–24, moderate risk 23–18, high risk ≤17). A blinded statistician will create a computerized random number generator, and then create blocks of six with intervention groups randomly assorted in each block (StataSE, Stata Corp, LLC, College Station, TX, United States). Other researchers and participants will not be blinded to intervention group, as this is not possible. However, participants will not be made aware of other intervention groups’ activities.

#### Intervention

There are two interventions and one control activity being compared in this study: prescribed use of minimal footwear, IFM strengthening exercises, and a sham intervention of seated active range of motion of the upper and lower extremities with no foot muscle involvement, accompanied by a brochure with home fall prevention recommendations (control activity). All intervention activities will be prescribed 5 days per week for 16 weeks. After the 16-week intervention period, participants will be encouraged to continue their intervention at least 2 days per week until the end of the 12-month study to maintain gains in muscle strength or endurance (American College of Sports Medicine et al., 2018). At least 2 days of continued training is recommended by the American College of Sports Medicine to maintain muscle strength and endurance (American College of Sports Medicine et al., 2018).

**FIGURE 3:**
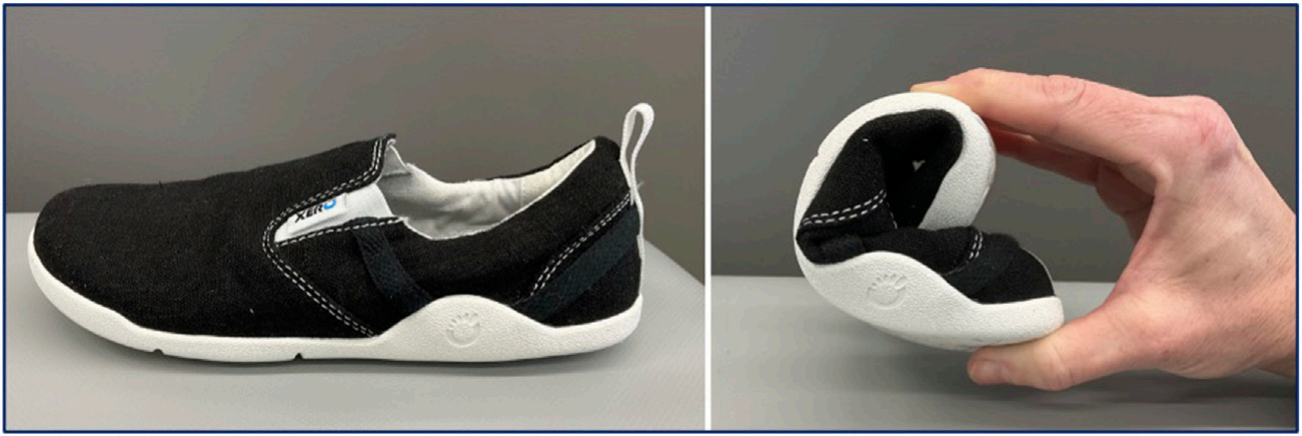
Xero Aptos minimal footwear.

Those in the minimal footwear group will be given a standardized pair of minimal shoes (Figure 3. Xero Aptos, Xero Shoes, Broomfield, CO, United States), and detailed instructions regarding gradual progressive use of the minimal footwear (Supplementary Material). In brief, the instructions include 30 min per day of continuous or non-continuous walking at a self-selected pace (using an assistive device as needed), and also wearing the footwear for progressively longer periods during daily activities.

Details and images of the IFM strengthening exercises can be found in Supplementary Material. They include seated lesser toe metatarsophalangeal joint (MTP) flexion/great toe MTP extension, great toe MTP flexion/lesser toe MTP extension, toe abduction, toe adduction, and the short foot exercise (McKeon et al., 2015; Glasoe, 2016; Gooding et al., 2016). These exercises will be prescribed as 3 sets of 10 repetitions for each movement, one time per day.

The control group will perform seated active range of motion activities for the upper and lower extremities, prescribed as 3 sets of 10 for each motion, one time per day. In addition, they will be given a home fall prevention brochure created by the Centers for Disease Control and Prevention (CDC, 2024), and instructed to review it on a weekly basis. These exercise activities will serve as a sham intervention as they do not in any way involve activation of the IFM and are not expected to have any meaningful effects on strength or balance. Details and images of the seated exercises and brochure can be found in Supplementary Material.

After the initial instruction session, instructors will meet with participants again at 1 week and 4 weeks to review intervention performance and answer questions.

In addition to the above interventions, all participants will be given a pedometer (3D Fit Bud, 3D Active, United Kingdom) and asked to record daily step count in a paper journal. This will allow for comparisons of general physical activity level of each group. Also in the paper journal, participants will be asked to record any falls and the details of the circumstances should a fall occur. To promote intervention adherence and to capture details of falls while fairly recent, participants will be contacted every 2 weeks by researchers from 4 weeks post-intervention instruction to the end of the 12-month participation period. Participants may choose their communication preference for these contacts (email, phone call, or text message). With each contact, five standardized questions will be asked, and a reminder given to not participate in other fall prevention programs while enrolled in the current study:

1. Have you experienced any falls (if yes, how many and can you provide details?)
2. Are you performing the study activities and do you have any questions or issues?
3. Are you using your journal?
4. Are you using the pedometer and recording daily step count?
5. Have you had any changes in health status?

All participants will be instructed in their intervention activities, pedometer use, and journal use by six trained Doctor of Physical Therapy (DPT) students. Two students will specialize in each group’s activities. Training for each group’s activities will include individual instruction and practice with the primary investigator, who is a physical therapist with 17 years of clinical experience. After instruction, practice, and individual feedback from the primary investigator, each DPT student will individually instruct a physical therapy faculty member unaffiliated with the study, and receive feedback. Written training materials for the DPT students are the same as the instructions that will be given to research participants and can be found in Supplementary Material.

#### Outcomes

At baseline, participants will be asked to self-report basic demographics (e.g., age, sex, height, weight, race, ethnicity, highest level of education), as well as history of falls in the past year, current use of an assistive device, level of pre-trial physical activity, and if they feel they spent a lot of their childhood and/or young adulthood barefoot. They will also have dominant hand grip-strength assessed in a seated position using a hand-held dynamometer. (Bohannon, 2019). A detailed medical history, including comorbid conditions and current medications will be collected through a clinical interview process similar to a physical therapy initial evaluation.

Measures of fall risk, proprioception, IFM cross-sectional area (cm^2^), navicular drop, and hallux valgus angle will be taken at baseline and 8-week, 16-week, and 12-month following intervention instruction. These measures will be conducted by two physical therapist researchers with assistance from DPT students. The physical therapist researchers will be blinded to treatment group; however, the DPT students will not be blinded. We have included details of each outcome measure and rationale for selecting the corresponding measurement instruments:

##### Fall risk using the Mini-BESTest (mini-balance evaluation systems test)

The Mini-BESTest is a clinical balance assessment tool that is a shortened version of the Balance Evaluation Systems Test (Franchignoni et al., 2010). It targets four different balance control systems that contribute to dynamic balance, which are considered functional and influenced by foot posture and proprioception (Menz et al., 2006). The four balance control systems are 1) anticipatory postural adjustments, 2) reactive postural control, 3) sensory orientation, and 4) dynamic gait; all of which may be affected by exercises targeting the IFM. There are 14 items scored on a 3-level ordinal scale. The maximum score is 28, indicating little to no fall risk, and the minimum score is 0. Based on a Rasch validation study by Franchignoni et al. (2015), we created three fall risk categories for participants’ total score (none to mild risk 28–24, moderate risk 23–18, high risk ≤17). The equipment required for the test includes a 4-inch foam pad (medium density T41 firmness rating), an incline board (10°–15°), a 9-inch-tall box, a chair without arm rests or wheels, a stopwatch, and a 3-m distance marked on the floor. It has been tested on several populations with balance disorders and was found to have high reliability and validity for measuring balance function and its change over time (Godi et al., 2013; O’Hoski et al., 2014). The Mini-BESTest has excellent concurrent validity with the Berg Balance Scale (r = 0.85, CI 0.78–0.90) (Franchignoni et al., 2010). It also has high content validity, as the test consists of other well-known balance tests including the Berg Balance Scale, the Timed Up and Go, the Dynamic Gait Index, and the Clinical Test of Sensory Integration in Balance (Franchignoni et al., 2010). The Mini-BESTest is cited as a good tool for predicting fall risk in community-dwelling older adults ages 60–102 (Magnani et al., 2020), and there are published normative values for healthy adults ages 50–89 (O’Hoski et al., 2014). When used to measure community-dwelling older adults with balance disorders, it had a low ceiling effect, an established minimal detectable change (MDC) of 3.5 points and cut-off scores for predicting falls based on age (Godi et al., 2013; O’Hoski et al., 2014; Marques et al., 2016). A minimal clinically important difference (MCID) of two points was established as significant progress in balance in adults with recent total knee arthroplasty, and authors recommended it as a balance assessment tool (Chan et al., 2020). The Mini-BESTest will be conducted by supervised DPT students and scored by a physical therapist researcher with 17 years of clinical experience. Further, each test will be video recorded for review and assistance with scoring.

DPT students will be trained in performing the Mini-BESTest by two physical therapy faculty members, one with over 30 years of clinical practice and teaching experience and the other with 17 years of clinical practice and 6 years of teaching experience. The Mini-BESTest is part of these students’ regular curriculum. They individually read and learn the components of the test along with the standardized script. They next practice the test on other students, and then practice the test on faculty members unaffiliated with the current research study. They also observe videos of older adults with different balance abilities partake in the Mini-BESTest.

##### Proprioception using the lower extremity position test (LEPT)

Proprioception is a sense or perception of position in an environment and employs a complex interaction of the visual, vestibular, and somatosensory systems (Rosker and Sarabon, 2010). It is a difficult element to measure given the number of structures that contribute to this sense, but from a clinically practical standpoint, the focus in this research will be on kinesthesia, or consciously perceived joint position sense. Literature regarding the reliability and validity of clinical ankle/foot proprioceptive measures is scarce. This body of literature tends to use highly technical measuring devices, oftentimes custom-made by researchers. A more simple and clinical approach to proprioception measurement can be done with active or passive positioning of a joint (Rosker and Sarabon, 2010). The LEPT is a passive positioning test that has recently been developed as a clinical tool without the use of technical equipment, and has been tested for reliability in healthy older adults (fair to good test-retest reliability, ICC = 0.60–0.68) and in populations post-stroke (good test-retest reliability ICC = 0.79–0.85) (Ofek, 2019; Ofek et al., 2019). This tool assesses a combined movement of the knee and ankle to two distances (12 and 22 cm) while the participant is in a seated position, eyes closed, wearing a thin sock, with their foot resting on a non-friction surface. The examiner slides the foot to a measured point on the floor (12 cm), returns it to the starting position, and then repeats the passive movement until the participant says “stop” at the point they think replicates the first position. The test is repeated using a 22 cm distance. The difference between the designated point and where the participant is asked to stop is calculated for each distance. Standard Error of Measurement (SEM) calculations from a reliability study of the LEPT by Ofek, (2019), found the 12 cm distance SEM to be 1.14 cm and the 22 cm distance SEM to be 1.28 cm. For the present study, measures within the SEM for each distance will be considered “accurate”. If a participant is outside the SEM, it will be considered “inaccurate”. A change from inaccurate to accurate performance will be considered an improvement in proprioception. Because there is evidence that IFM strengthening interventions can affect loading and alignment of the knee (Trombini-Souza et al., 2015), and because previous research participants’ subjective reports suggested enhanced overall lower extremity proprioception (Franklin et al., 2017b), it seems practical to include a proprioceptive measure that may capture effects beyond the foot and ankle. A physical therapist researcher with 17 years of clinical experience will conduct this measure.

**FIGURE 4:**
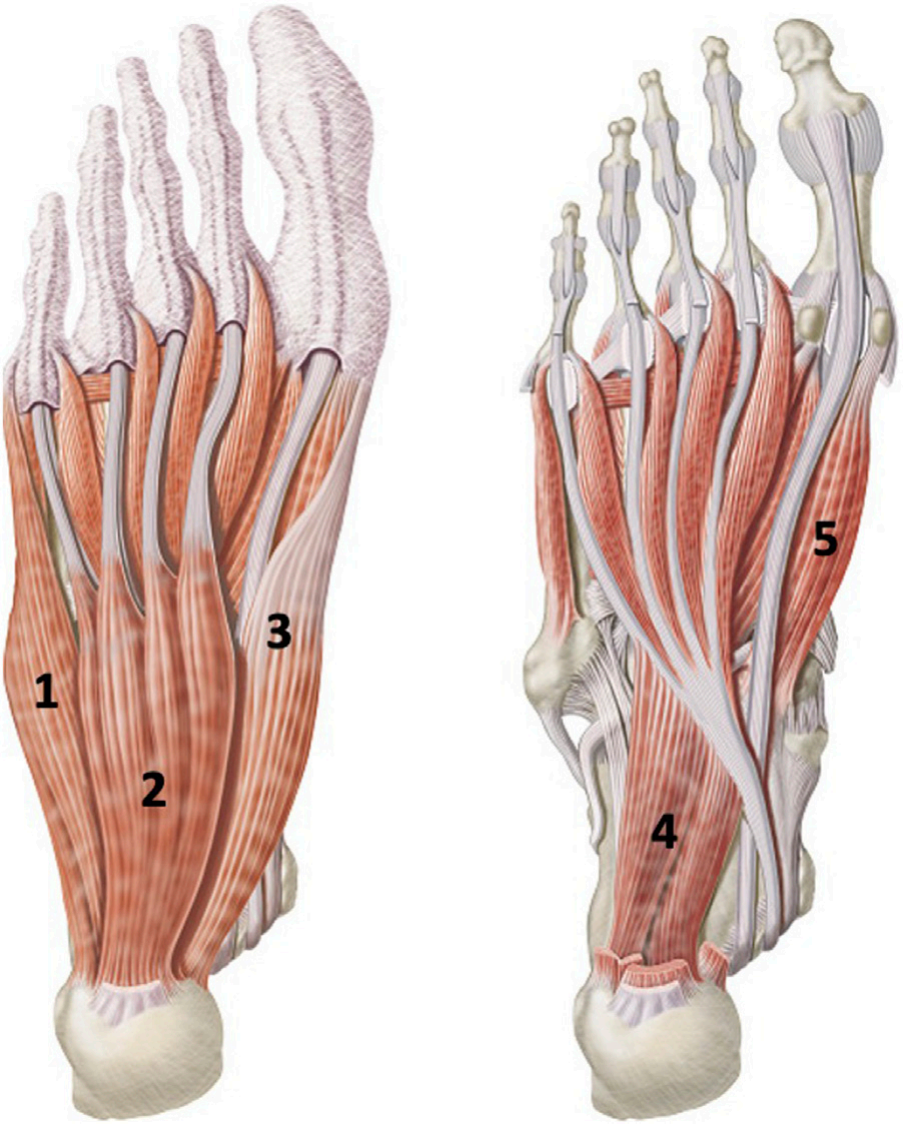
Five plantar intrinsic foot muscles measured by ultrasound imaging. 1. abductor digiti minimi, 2. Flexor digitorum brevis, 3. Abductor hallucis, 4. Quadratus plantae, and 5. Flexor hallucis brevis. Images purchased via iStock by Getty Images, Calgary, Alberta Canada.

Intrinsic foot muscle cross-sectional area (CSA, cm2) of the right abductor hallucis, flexor hallucis brevis, flexor digitorum brevis, quadratus plantae, and abductor digiti minimi muscles will be measured using ultrasound imaging (Figure 4). Although there are additional plantar intrinsic foot muscles, these five muscles were selected based on existing research regarding ultrasound imaging measurement reliability, and based on links to foot function. The decision to measure only one foot (right) is due to the number of participants (n = 90), time points (n = 4), and muscles (n = 5) totaling 1800 data points related to muscle morphology alone. Ultrasound imaging is a reliable method to measure morphology of IFM in young and middle-aged adults as shown by previous researchers (McKeon et al., 2015; Fraser and Hertel, 2019a; Mickle et al., 2013; Crofts et al., 2014; Latey et al., 2018; Fraser et al., 2018; Wong, 2007). In these previous studies, researchers measured IFM muscle thickness or cross-sectional area and found good to excellent between-session and inter and intra-operator reliability. In brief, for this study, images of each muscle will be captured at a frequency of 10 MHz with a GE NextGen LOGIQ e R7 ultrasound unit (GE Healthcare, United States) with a wide-band linear array (12 L-RS) probe using techniques described by Latey et al. (2018) and Mickle et al. (2013) With the participant positioned in right side-lying on a height-adjustable treatment table, each muscle will be located using adjacent bony landmarks, then viewed in the long axis. Passive and active motion of the corresponding toe(s) will be performed to confirm the muscle. The thickest portion of each muscle will be visually identified, centered on the monitor, and then the probe will be rotated 90° to obtain a short-axis view. Passive and active movements again can be used to confirm the muscle as needed, and an image will be captured. Two images of each muscle will be captured using this same technique. A single physical therapist researcher will perform all ultrasound imaging. Intra-reliability analyses using intraclass correlation coefficients (ICC) and Fleiss’ kappa will be conducted to confirm accuracy of this measure. Values will be interpreted as 0–0.5 poor, 0.51–0.74 fair, 0.75–0.9 good, and >0.9 excellent reliability, with ≥0.75 being acceptable agreement. A separate researcher blinded to intervention group will view each captured image and determine the CSA using ImageJ software (National Institutes of Health). For each muscle, the two CSA measures will be averaged and analyzed for changes in size. An increase in CSA of a muscle indicates hypertrophy, and thus, increased strength (Mayer et al., 2011). Calculations of muscle size change will be presented as percent changes.

##### Foot structure using navicular drop for foot pronation and hallux valgus angle (first metatarsophalangeal joint)

Navicular drop is a simple and clinically applicable measure of foot pronation requiring only a ruler. It involves measuring the height of the navicular bone from the floor when a participant is seated with the hip and knee at 90° and the foot and ankle positioned in subtalar joint neutral. The participant then moves to bilateral full weight bearing relaxed stance, and the height of the navicular bone is re-measured. The difference is taken between the seated and relaxed stance measures. A normal navicular drop is ≤10 mm while excessive and possibly pathological navicular drop is ≥15 mm (Brody, 1982). We will use these values to indicate improvement in arch height following interventions. Navicular drop has been found a reliable measure for foot pronation by several authors when performed by an experienced clinician (Menz, 1998). Excessive foot pronation is dysfunctional, can lead to several foot disorders and can contribute to falls in older adults (Menz et al., 2006; Hagedorn et al., 2013).

Hallux valgus angle is a simple clinical measure performed with a handheld goniometer with the participant seated with the hip, knee, and ankle joints at 90° and the foot resting on the floor. Hallux valgus is defined as ≥15° of abduction of the hallux from the first metatarsal, and has been further classified into stages of severity (mild 15°–25°, moderate 25°–35°, severe >35°) (Hagedorn et al., 2013). Greater hallux valgus angle has been linked to fall risk by several authors (Menz et al., 2006; Mickle et al., 2009; Koski et al., 1996). Measurement of hallux valgus is typically included in a comprehensive foot exam and was found to have good inter examiner reliability in a population of older adults (kappa values > 0.85, p < 0.01). (Hagedorn et al., 2013). When compared to the gold standard of radiographic measures of hallux valgus, clinical goniometry performed by an experienced clinician was within one°–5.5° (Janssen et al., 2014). Tang et al. conducted a 3 month intervention to reduce hallux valgus angle and found a reduction of 6.5° ± 3.8 resulted in significantly reduced pain and improved walking ability (Tang et al., 2002). Based on Tang et al.‘s work, we will consider a change of ≥7° to be a meaningful improvement. Although IFM function contributes to foot and toe alignment, we have included hallux valgus as an exploratory measure and do not anticipate it will significantly change in an older adult population. This can be likened to the malalignment of an osteoarthritic knee that does not change with exercise intervention alone (Raghava et al., 2001).

A physical therapist researcher with 17 years of clinical experience will perform all foot measures.

Fall Rate will be determined by the number of falls per person in the 12-month study period. Falls will be recorded based on journal entries and will be cross-referenced with subjective reports at 2-week contacts. Researchers and participants will use the operational definition of a fall as an unexpected event in which a person comes to rest on the ground or lower level (Laurence, 2021; Guirguis-Blake et al., 2018).

#### Qualitative and implementation data

At the 16-week measurement point, a semi-structured interview will be conducted with each participant and recorded for later analysis. The purpose of the interview will be to gain understanding of the research activities themselves to inform future studies and also to gain subjective reports of the effects of each intervention. In addition, the journals participants keep regarding adherence to the interventions may inform future research. We will collect participant impressions and level of difficulty regarding performance of their intervention or control activity, comfort of the footwear (for footwear group), pedometer use, adherence to study activities, journal use, and subjective reports on their intervention’s (or control’s) effects on foot awareness (layman’s term for kinesthesia), influence on pre-existing pain or orthopedic conditions, and on balance.

Participants will rate their impressions of intervention or control difficulty on a 5-point Likert scale (e.g., 0 = no difficulty, 1 = mild difficulty, 2 = moderate difficulty, 3 = severe difficulty, 4 = unable to perform). In addition, the semi-structured interview will allow for qualitative data collection regarding participant impressions beyond the Likert scale scores. For example, if a participant has poor adherence to an intervention, follow up questions regarding barriers and facilitators may be valuable in guiding future research or clinical application. Participant impression data may be analyzed to compare participants who drop out of the study *versus* those who complete all interventions and measurement sessions. And finally, adverse events such as pain, injury, or safety concerns will be recorded. An example of a semi-structured interview script can be found in Supplementary Material. The interviews were made as similar as possible for each intervention group.

#### Data collection and management

Participant intake data, foot structure measures, proprioception measures, and Mini-BESTest fall risk measures will be collected on paper intake forms and transferred to a password protected web-based data storage system utilizing spreadsheets. Ultrasound images will be recorded and stored in the ultrasound unit, as well as loaded to a USB thumb drive and transferred to the password protected web-based data storage system. The ultrasound unit, thumb drive, and paper forms will be kept in a locked office with access only to key research personnel. The videos of the Mini-BESTest measures will be uploaded to the password protected web-based data management system and then deleted from the recording device. All data will be de-identified and labeled using an alphanumeric system. Participant contact information and medical history with identifiable information will be kept in a password protected file in the web-based system and in a locked office with access only to key research personnel.

#### Statistical analysis

Fall risk (MiniBESTest scores), muscle CSA of five IFM, Lower Extremity Position Test scores, navicular drop, and hallux valgus angle data will be compared using a *three-group x four-time points* repeated measures analysis of covariance (ANCOVA).

Fall rate will be measured using the number of falls per person in a 12-month period, beginning with the date of the baseline measure. A Cox proportional hazards model will be used to compare falls across groups.

Demographic and potential confounding variables will be collected and included in analyses (e.g., age, sex, height, weight, race, ethnicity, education, pre-trial physical activity (quantified based on the Physical Activity Guidelines for Americans, older adults) (Piercy et al., 2018), pre-trial falls history, comorbidities, medications, self-reported visual impairment, and grip strength).

Implementation variables will be analyzed quantitatively via adherence records and Likert scales, as well as qualitatively via semi-structured interviews.

Our primary analytic approach is an intention to treat analysis. We also will conduct a per protocol analysis for participants who do not complete all study activities (missing data). This may include analysis for mechanisms of missingness: missing completely at random (MCAR), missing at random (MAR) or missing not at random (MNAR). Based on the mechanism of missingness, we may perform a listwise or case deletion or last observation carried forward if appropriate. (Kang, 2013).

### Anticipated results

We hypothesize that both IFM strengthening exercises and prescribed minimal footwear use will result in reduced fall risk exceeding the established MDC (3.5 points) or MCID (2 points), improved proprioception, and increased IFM size (CSA), and will result in no change in foot structure measures. Those participants performing IFM strengthening exercises will likely have greater changes in muscle size than those using minimal footwear due to the intentional recruitment of the IFM. However, the changes in fall risk and proprioception will likely not differ significantly between minimal footwear use and IFM strengthening exercise groups. We hypothesize no changes will occur with fall risk, proprioception, IFM size, and foot structure in the control group.

It is unknown how our cohort of older adults will receive the IFM strengthening exercises or minimal footwear, but based on limited evidence in nations outside the United States, some older adults can successfully perform these interventions. Our study will be conducted in a region with cold weather, which could limit adherence to minimal footwear use. We plan to encourage participants to use footwear in large indoor spaces such as shopping malls. Severe foot and toe stiffness could make performance of the IFM strengthening exercises difficult for some individuals. We do not anticipate any limitations for the control (sham intervention) group.

Several factors have been included in the study design in an effort to keep participants engaged in the study for 12 months. We have seven in-person meetings planned for measurement and intervention instruction and review. In addition, each participant will have two specific Doctor of Physical Therapy students assigned to them for intervention instruction, intervention review, scheduling, and bi-monthly contacts. This personal connection may keep participants adherent and engaged in study activities.

## Discussion

Strengthening of the IFM in older adults may lead to a reduction of falls and injuries in this population, but this is not yet established. The aims for this study address multiple gaps in the literature. First, this study can improve our knowledge of the IFMs’ role in balance and mobility in older adults. Second, this study seeks to establish an effective approach to improving IFM strength in older adults. Evidence is limited regarding the best interventions by which to strengthen and/or enhance proprioceptive abilities of foot structures, specifically the IFM (Futre et al., 2022). The function of the IFM in terms of proprioceptive or motor control qualities is largely speculative and has not been objectively measured in older adults (Futre et al., 2022). A recent systematic review of IFM-related studies (Jaffri et al., 2023) found only two studies that objectively measured effects on sensory or motor control function. One study found improvements in proprioception and balance following IFM strengthening; however, participants in this study were 19–29 years old (Lee et al., 2019). The other found improved motor performance with IFM exercises after a 4-week intervention, with a mean age of participants of 21.5 years (Fraser and Hertel, 2019a). To date, there are no known investigations regarding proprioceptive, sensory, or motor control effects of IFM strengthening interventions in older adults. Subjectively, older adults who have performed IFM strengthening interventions report improved foot and/or lower extremity awareness and improved balance (Futre et al., 2022). This suggests an important somatosensory contribution of the IFM that is largely unexplored in older adults. Finally, isolated IFM strengthening interventions may have an impact beyond the foot. The local changes in foot strength and/or proprioception may lead to improved balance and functional mobility, reduced falls, and promotion of greater physical activity in older adults (Futre et al., 2022).

Physical therapists and other healthcare providers could incorporate IFM strengthening interventions into rehabilitation, fall prevention, and physical activity programs, potentially by wearing minimally cushioned footwear during exercise or daily activities, or by teaching older adults how to safely perform and integrate specific IFM strengthening exercises into their routine. These interventions could be conducted at a community-wide level at senior centers, assisted living facilities, or group exercise classes that already promote fall-prevention activities. However, before we can widely apply these interventions for general health and fall prevention purposes, we must understand their mechanisms and have evidence to guide their implementation. For example, there is little to no guidance on how to safely transition from traditionally cushioned shoes to minimal footwear (Futrell et al., 2024). Going from cushioned supportive shoes that essentially brace the foot, to a shoe that promotes foot mobility, places new demands on musculoskeletal structures. Just as with any activity that places new demands on muscles and joints, gradual and progressive load is necessary to prevent injury. The same can be said for specific IFM strengthening exercises. Improper technique could lead to joint alignment faults or muscle substitutions. Therefore, evidence is necessary to guide providers on how to best implement these promising interventions for their older adult patients and clients. When performed correctly, these are simple, safe, and affordable interventions that could have a major influence on older adults’ fall risk, mobility, and independence.

The use of ultrasound imaging in physical therapy research and practice has only recently become common. Clinical testing of IFM strength is difficult to isolate from the extrinsic foot muscles. Those methods are in development, but have not been established (Bruening et al., 2019; Ridge et al., 2017). Ultrasound imaging is the only method with which to directly measure changes in IFM size, and therefore, can be used as an indirect measure for increased strength. An increase in muscle size (hypertrophy) implies an increase in muscle strength. Ultrasound imaging of the IFM has been performed in research regarding young and middle-aged adults, and has also been performed successfully in at least one observational study involving older adults (Mickle et al., 2016a).

Aside from the measurement approach, the topic of IFM research in general is innovative as it has only recently begun building a body of evidence. This is especially true for an older adult population. Much of the information regarding the *function* of the IFM is speculative and has not been formally studied. We know very little about these muscles in younger or older adults, but the research that exists shares the common theme that these muscles are important because they are the first point of contact between our bodies and the environments we navigate.

### Potential limitations and solutions

We acknowledge a few limitations of this study, with possible solutions or mitigations.

Falls are multifactorial and caused by internal and external factors. The specific inclusion and exclusion criteria of this study attempts to minimize many internal confounding factors including poor foot sensation, vestibular disorders, cognitive impairments, amputations, and recent injury or surgery.

The participants will be asked to self-report falls, details of falls, intervention adherence, and step count in a paper journal. We recognize that self-reported data can be inaccurate or unreliable; however, we have mitigated this with a plan to contact participants every 2 weeks as a reminder and a supplemental way to collect this same data.

Our decision to collect foot measures using only the right foot is one based on time and resources for both researchers and participants. We recognize people can have asymmetry in the feet in terms of foot dominance, structure or pathology. Having only right foot measures limits the generalizability of our results.

The study will be conducted in an area of the United States with extremely cold winter weather. This may limit some participants’ ability to engage in the prescribed walking/wearing schedule of the minimal footwear. We will recommend people walk in minimal footwear in safe indoor spaces such as grocery stores or shopping malls, but accessibility to those spaces may be limited based on socioeconomic status. In addition, it is possible that participants wearing minimal footwear will be more unstable initially compared to their customary footwear. The walking/wearing schedule takes this into account with a very conservative increase in wear time to allow for the musculoskeletal and neuromuscular systems to gradually adapt to the footwear’s wider, thinner design.

This study addresses the very substantial clinical and community health issue of falls in older adults. The overall design and attention to detail will produce high quality research that may provide researchers, clinicians, policymakers, and older adults themselves with simple solutions to this highly relevant problem.

## Data Availability

Data is not yet publicly available. Data requests for this project can be made here: efutrell@springfieldcollege.edu.
