# Supplementary material for: The effects of intrinsic foot muscle strengthening interventions for adults over age 65: a randomized controlled trial protocol
Source: Front Aging. 2025 Oct 15;6:1622232. doi: 10.3389/fragi.2025.1622232 (PMC12568628; doi:10.3389/fragi.2025.1622232)

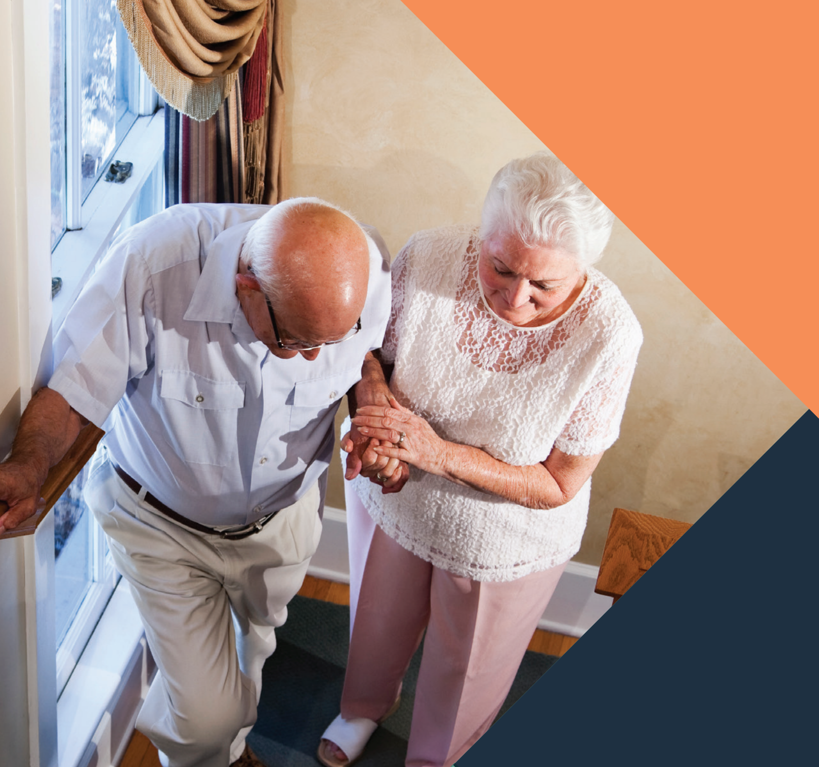

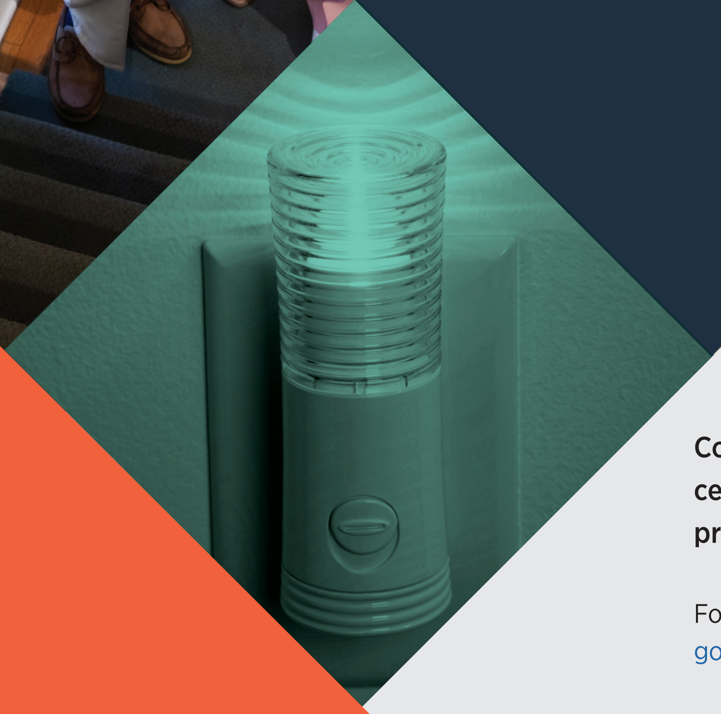

Contact your local community or senior center for information on exercise, fall prevention programs, or options for improving home safety.

For additional information on fall prevention, visit go.usa.gov/xN9XA

Centers for Disease Control and Prevention
National Center for Injury Prevention and Control

2017

Check for Safety

A Home Fall Prevention Checklist for Older Adults

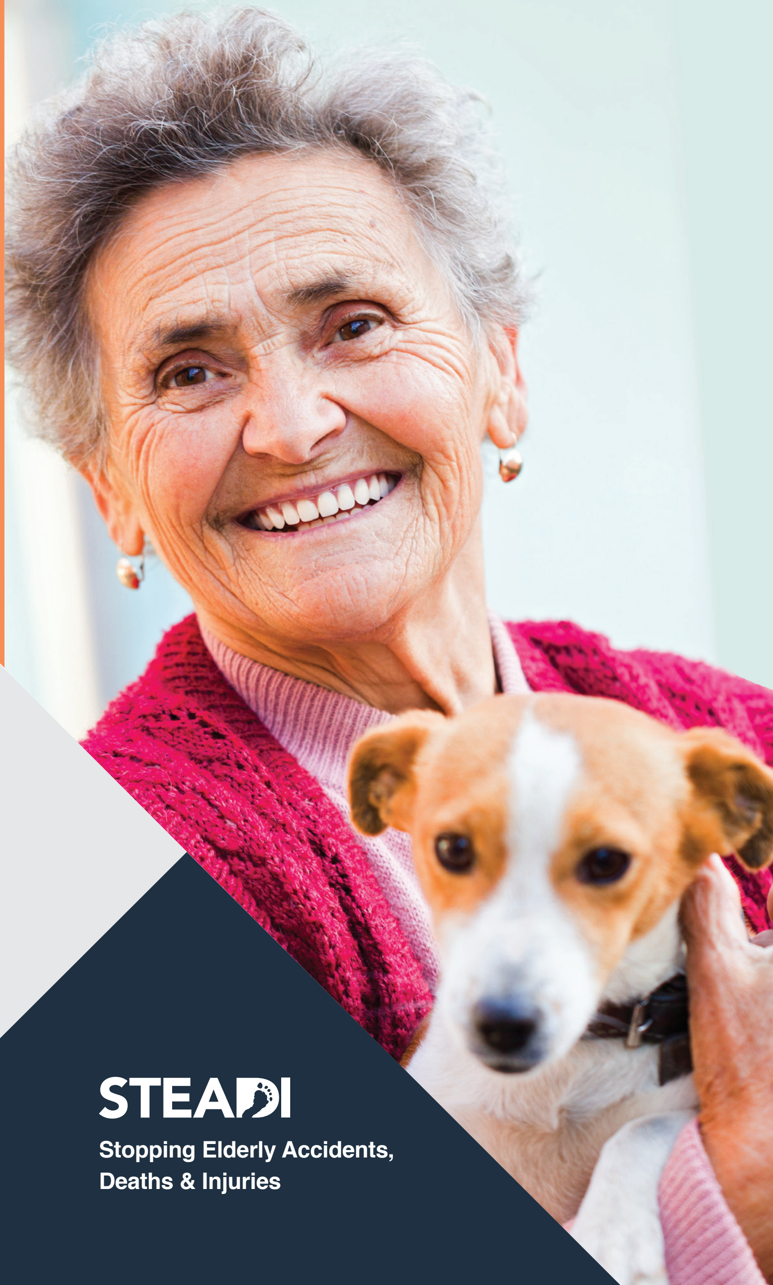

STEADI

Stopping Elderly Accidents, Deaths & Injuries

Use this checklist to find and fix hazards in your home.

STAIRS & STEPS (INDOORS & OUTDOORS)

Are there papers, shoes, books, or other objects on the stairs?

- ☐ Always keep objects off the stairs.

Are some steps broken or uneven?

- ☐ Fix loose or uneven steps.

Is there a light and light switch at the top and bottom of the stairs?

- ☐ Have an electrician put in an overhead light and light switch at the top and bottom of the stairs. You can get light switches that glow.

Has a stairway light bulb burned out?

- ☐ Have a friend or family member change the light bulb.

Is the carpet on the steps loose or torn?

- ☐ Make sure the carpet is firmly attached to every step, or remove the carpet and attach non-slip rubber treads to the stairs.

Are the handrails loose or broken? Is there a handrail on only one side of the stairs?

- ☐ Fix loose handrails, or put in new ones. Make sure handrails are on both sides of the stairs, and are as long as the stairs.

FLOORS

When you walk through a room, do you have to walk around furniture?

- ☐ Ask someone to move the furniture so your path is clear.

Do you have throw rugs on the floor?

- ☐ Remove the rugs, or use double-sided tape or a non-slip backing so the rugs won't slip.

Are there papers, shoes, books, or other objects on the floor?

- ☐ Pick up things that are on the floor. Always keep objects off the floor.

Do you have to walk over or around wires or cords (like lamp, telephone, or extension cords)?

- ☐ Coil or tape cords and wires next to the wall so you can't trip over them. If needed, have an electrician put in another outlet.

KITCHEN

Are the things you use often on high shelves?

- ☐ Keep things you use often on the lower shelves (about waist high).

Is your step stool sturdy?

- ☐ If you must use a step stool, get one with a bar to hold on to. Never use a chair as a step stool.

BEDROOMS

Is the light near the bed hard to reach?

- ☐ Place a lamp close to the bed where it's easy to reach.

Is the path from your bed to the bathroom dark?

- ☐ Put in a nightlight so you can see where you're walking. Some nightlights go on by themselves after dark.

BATHROOMS

Is the tub or shower floor slippery?

- ☐ Put a non-slip rubber mat or self-stick strips on the floor of the tub or shower.

Do you need some support when you get in and out of the tub, or up from the toilet?

- ☐ Have grab bars put in next to and inside the tub, and next to the toilet.

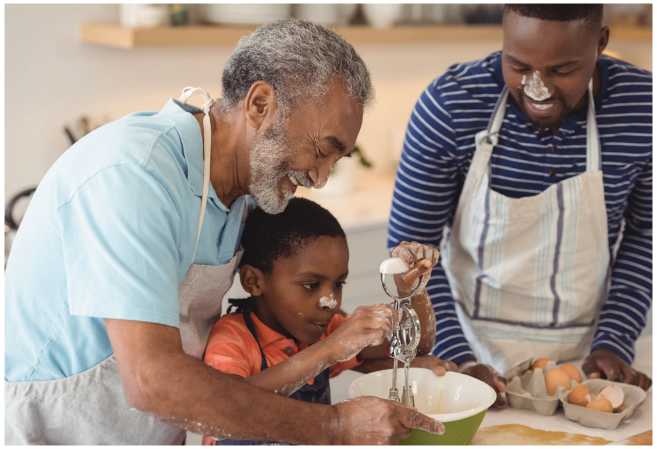

Supplement: Supplementary file 1 [file Supplementaryfile5.pdf]
