# Supplementary material for: The effects of intrinsic foot muscle strengthening interventions for adults over age 65: a randomized controlled trial protocol
Source: Front Aging. 2025 Oct 15;6:1622232. doi: 10.3389/fragi.2025.1622232 (PMC12568628; doi:10.3389/fragi.2025.1622232)
Supplement: Supplementary file 2 [file Supplementaryfile1.docx]

**Footwear and Walking Instructions**

Because these are new shoes and your body is likely not accustomed to this type of footwear, you must wear them **gradually** at first.

The goal is to **intentionally walk in the Xero shoes for 30 minutes per day**, and eventually to wear the shoes for longer periods of time during regular daily activities. We have attached a schedule of how to do this.

For the next 4 months:

- Please follow the attached schedule **5 days per week** for walking in and wearing your Xero shoes
- Record your performance in your Daily Footwear Use and Falls Journal

Your start date is ___________________ The end of your 4-months date is __________________

After the 4 months date, please continue using the shoes as prescribed at least 2 days per week until your end date.

Your study end date is _____________________.

We will see you in person 1 week (date ________) and 4 weeks (date_______) after you start this program. At those times we can answer any questions, and we will observe your walking and the shoes’ structure.

We will also contact you by phone, text, or email (your preference) every 2 weeks throughout the study.

We will perform in-person measurement sessions at week 8________, week 16_________, & 1 year_______

Xero shoe walking and daily use schedule. Follow these instructions **5 days per week for 4 months (16 weeks)**.

| Dates | | | | Instructions | |
| --- | --- | --- | --- | --- | --- |
| Weeks 1 & 2 | | Starts on this date:  Ends on this date: | | While wearing the Xero shoes: go for a 10-minute walk at 3 separate times each day.  If you must rest during your walks, that’s fine, but be sure to count your **actual walking minutes for a total of 30.**  Total time in shoes each day is 30 minutes. | |
| Weeks 3 & 4 | | Starts on this date:  Ends on this date: | | While wearing the Xero shoes: go for one 10-minute walk and one 20-minute walk each day.  If you must rest during your walks, that’s fine, but be sure to count your **actual walking minutes, for a total of 30.**  Also wear your shoes for 1 hour of daily activities.    Total time in shoes each day is 1 hour and 30 minutes. | |
| Weeks 5 & 6 | Starts on this date:  Ends on this date: | | You may now wear the Xero shoes continuously for 30 minutes of walking. If needed, the walking can be broken into separate times. If you must rest during your walks, that’s fine, but be sure to count your **actual walking minutes, for a total of 30.**  Also wear your shoes for 2 hours of daily activities  Total time in shoes each day is 2 hours and 30 minutes. | |  |
| Week 7 | Starts on this date:  Ends on this date: | | Continue to wear the Xero shoes for 30 minutes of walking. If needed, the walking can be broken into separate times. If you must rest during your walks, that’s fine, but be sure to count your **actual walking minutes, for a total of 30.**  Also wear your shoes for 3 hours of daily activities.  Total time in shoes each day is 3 hours and 30 minutes. | |  |
| Week 8 | Starts on this date:  Ends on this date: | | Continue to wear the Xero shoes for 30 minutes of walking. If needed, the walking can be broken into separate times. If you must rest during your walks, that’s fine, but be sure to count your **actual walking minutes, for a total of 30.**  Also wear your shoes for 4 hours of daily activities.  Total time in shoes each day is 4 hours and 30 minutes | |  |
| Week 9 | Starts on this date:  Ends on this date: | | Continue to wear the Xero shoes for 30 minutes of walking. If needed, the walking can be broken into separate times. If you must rest during your walks, that’s fine, but be sure to count your **actual walking minutes, for a total of 30.**  Also wear your shoes for 5 hours of daily activities.  Total time in shoes each day is 5 hours and 30 minutes | |  |
| Week 10 | Starts on this date:  Ends on this date: | | Continue to wear the Xero shoes for 30 minutes of walking. If needed, the walking can be broken into separate times. If you must rest during your walks, that’s fine, but be sure to count your **actual walking minutes, for a total of 30.**  Also wear your shoes for 6 hours of daily activities.  Total time in shoes each day is 6 hours and 30 minutes | |  |
| Weeks 11-16 | starts on this date:  ends on this date: | | Continue to wear the Xero shoes for 30 minutes of walking. If needed, the walking can be broken into separate times. If you must rest during your walks, that’s fine, but be sure to count your **actual walking minutes, for a total of 30.**  Also wear your shoes for 7 hours of daily activities.  Total time in shoes each day is 7 hours and 30 minutes | |  |
| Weeks 17-52 |  | | For the remainder of the study **at least 2 days per week**:    Wear the shoes during daily activities for at least 6 hours AND wear the shoes while doing at least 30 minutes of intentional walking. | |  |
