# Supplementary material for: The effects of intrinsic foot muscle strengthening interventions for adults over age 65: a randomized controlled trial protocol
Source: Front Aging. 2025 Oct 15;6:1622232. doi: 10.3389/fragi.2025.1622232 (PMC12568628; doi:10.3389/fragi.2025.1622232)
Supplement: Supplementary file 3 [file Supplementaryfile3.docx]

**Exercise Instruction and Falls Brochure**

For the next 4 months:

- Please do each of these **exercises** **1 time per day, 5 days per week**
- Read the **brochure** and make any possible adjustments to your home **1 time per week**
- Record your performance in your Daily Exercise and Falls Journal

Your start date is ___________________ The end of your 4-months date is __________________

After the 4 months date, please continue the exercises 2 days per week until your end date.
Continue to use the brochure to check your home for fall hazards 1 time per month until your end date.

Your study end date is _____________________.

We will see you in person 1 week (date_________) and 4 weeks (date_________) after you start this program. At those times we can answer any questions, and we will observe your technique with the exercises.

We will also contact you by phone, text, or email (your preference) every 2 weeks throughout the study.

We will perform in-person measurement sessions at week 8________, week 16________, & 1 year_______.

Follow these instructions **5 days per week for 4 months (16 weeks)**.

| 1. **Seated upper extremity “X”**  - Start with palms together, elbows straight, hands at chest level. - Separate arms in a diagonal line as though making half of an “X”.  Repeat this 5 times. - Do the opposite direction as though making the other half of the “X”. Repeat 5 times.   After 5 repetitions in each direction, rest for 10 seconds.  Perform 3 sets  (Total = 30 arm movements) | 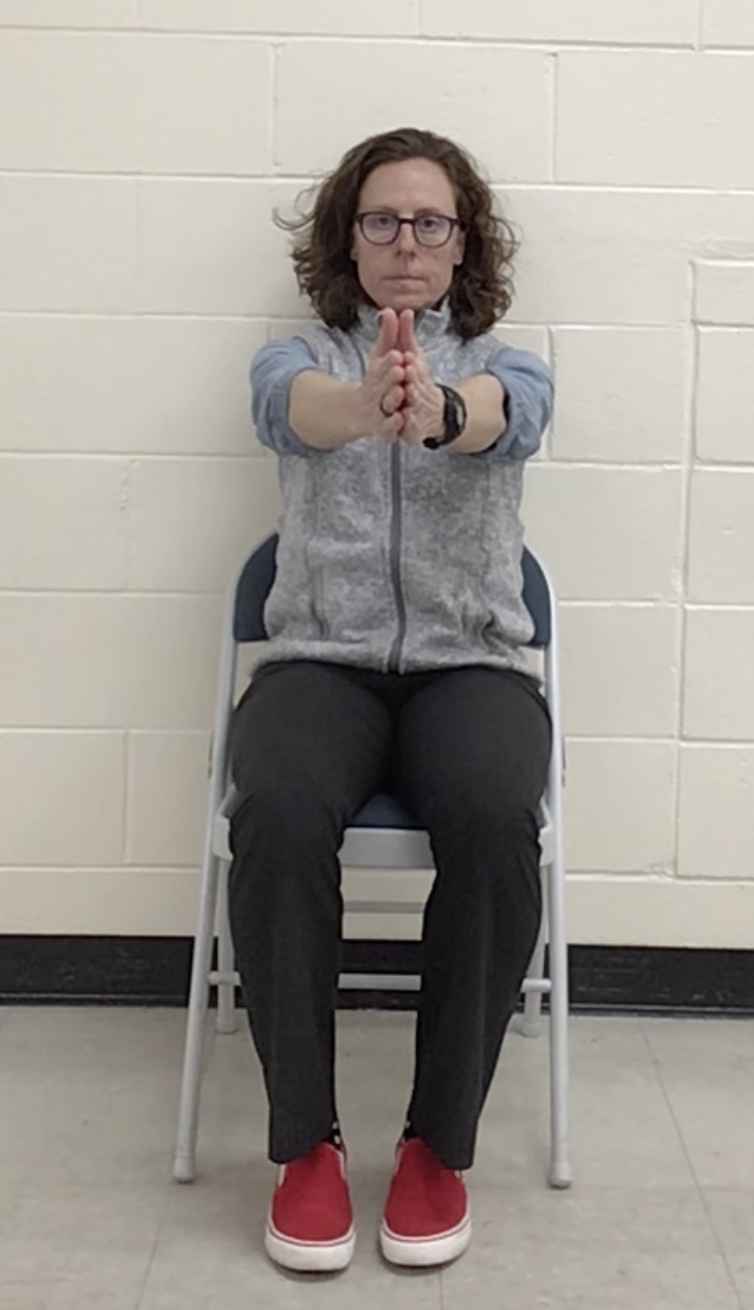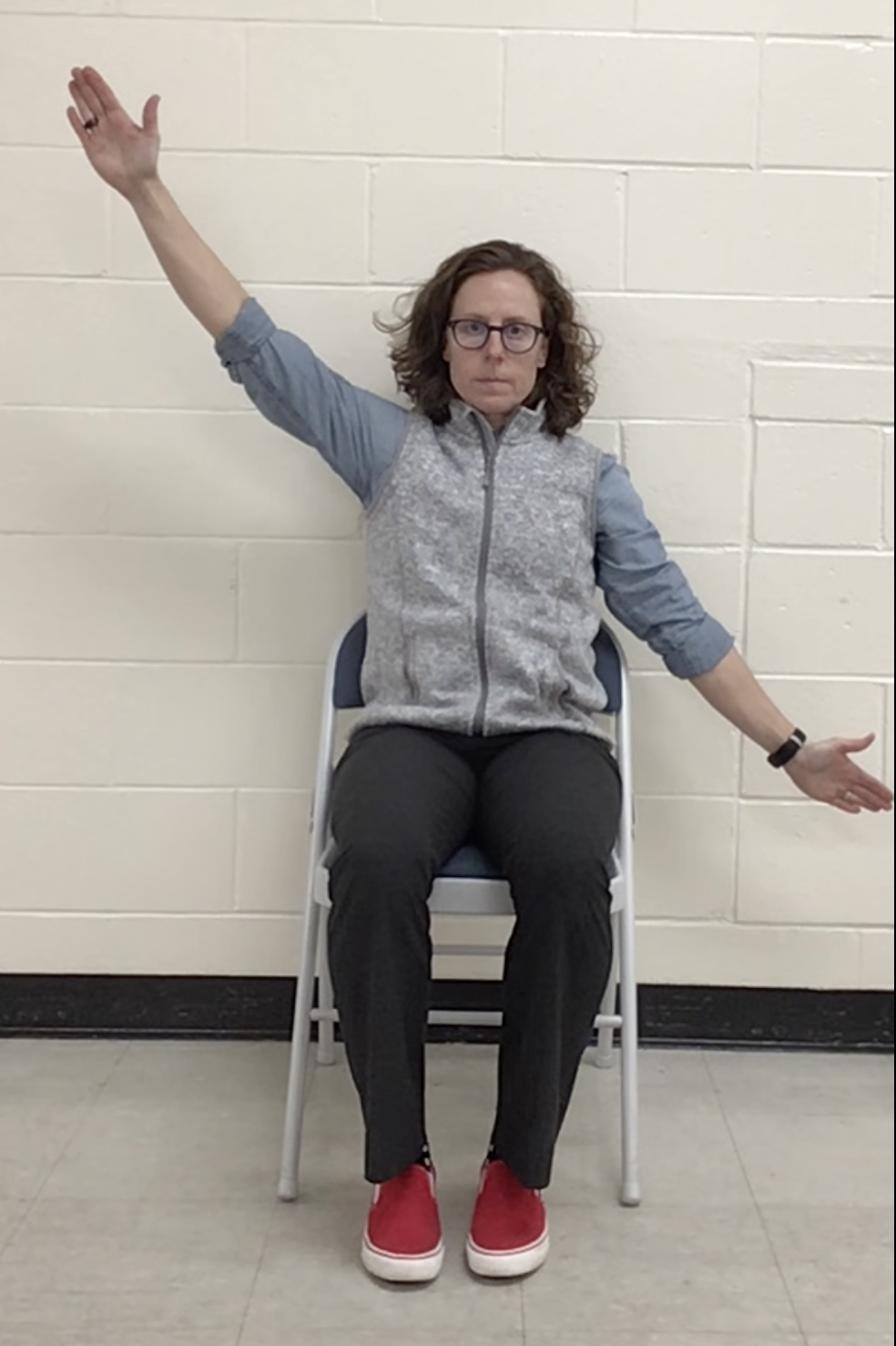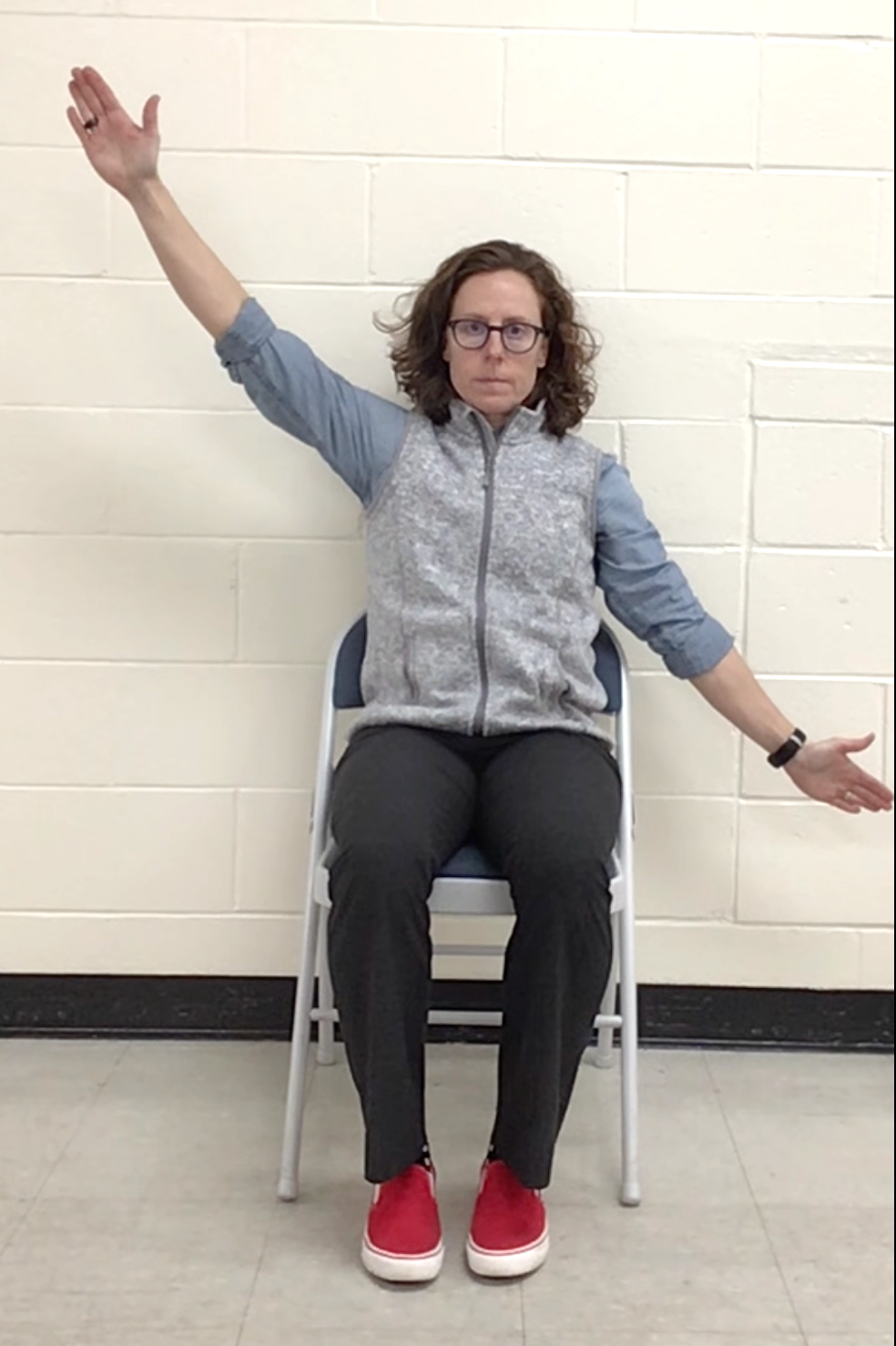  Right arm up x 5  Left arm up x 5  Starting position |
| --- | --- |
| 1. **Seated marching**  - Sit upright in chair with knees bent. - March in place, lifting each foot a few inches off the ground. - Repeat 10 marching steps, then rest for 10 seconds.   Perform 3 sets (Total = 30 marching steps) | 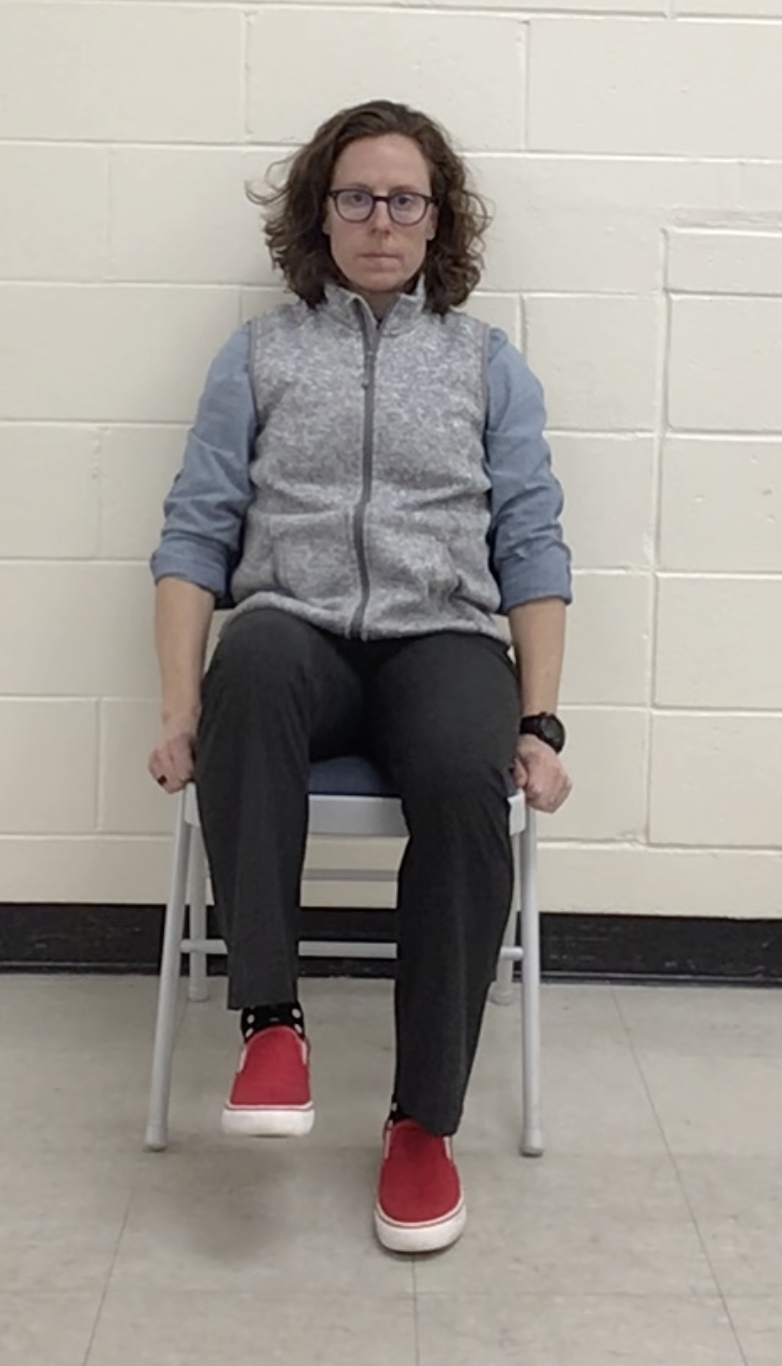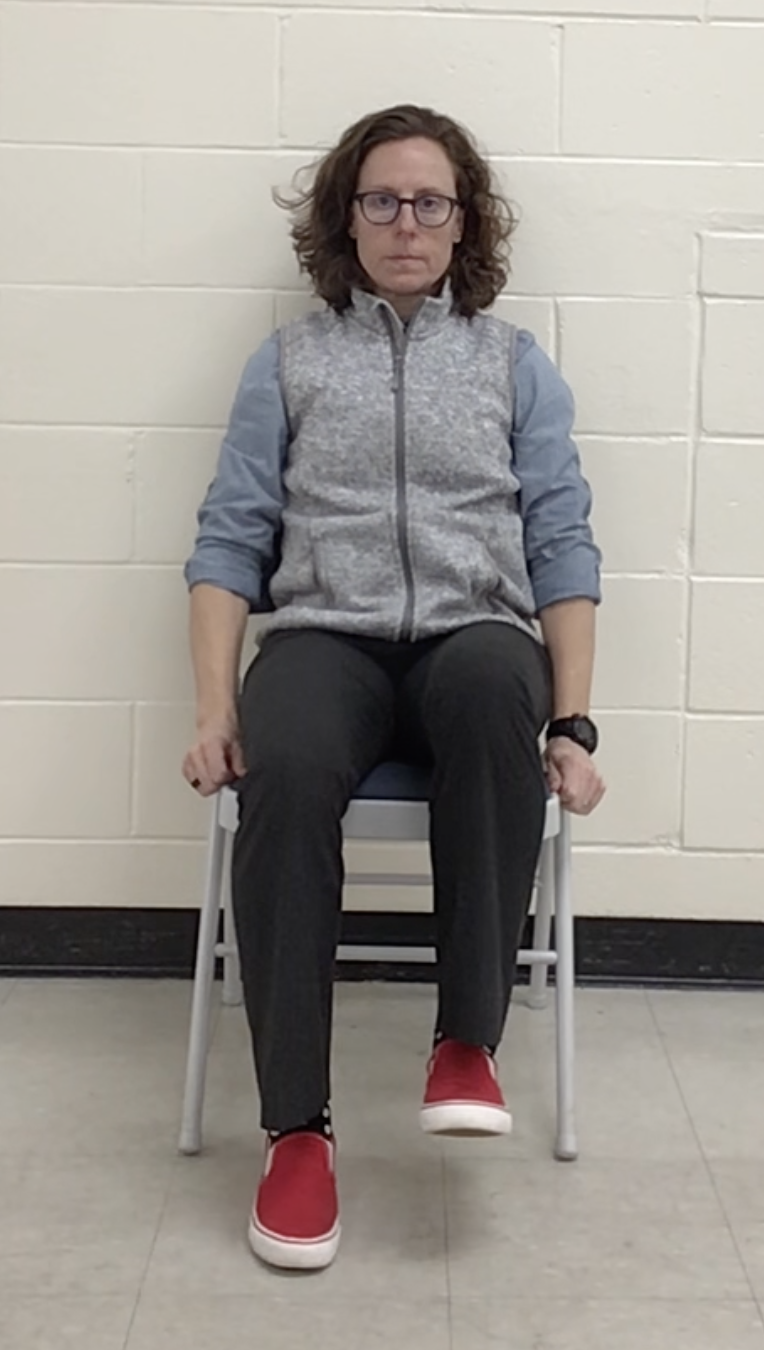 |
| 1. **Seated knee extension**  - Sit upright with knees bent. Straighten one knee by lifting the heel off the ground. - Perform 10 repetitions on the right, then rest 10 seconds and perform 10 repetitions on the left.   Perform 3 sets on each leg  (Total =30 on each leg) | 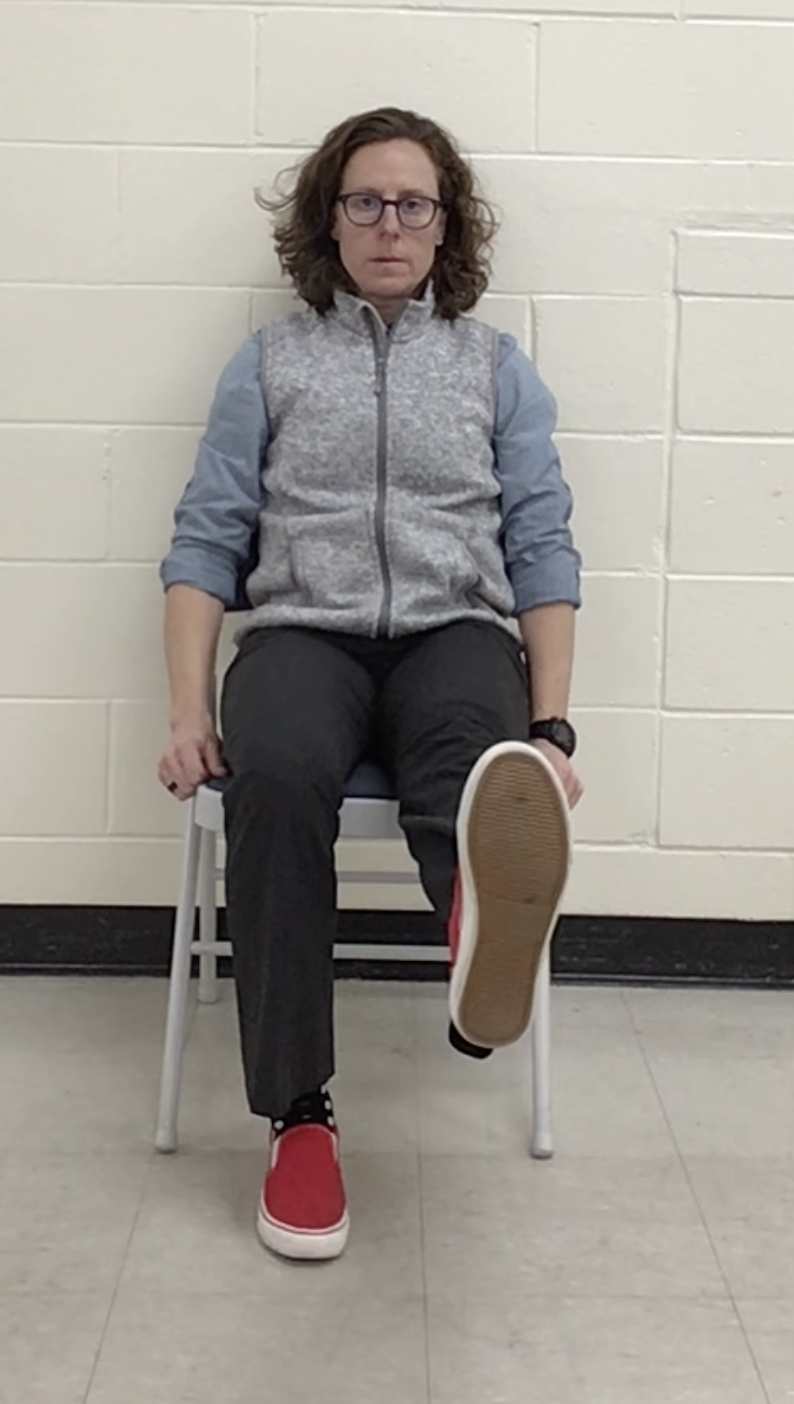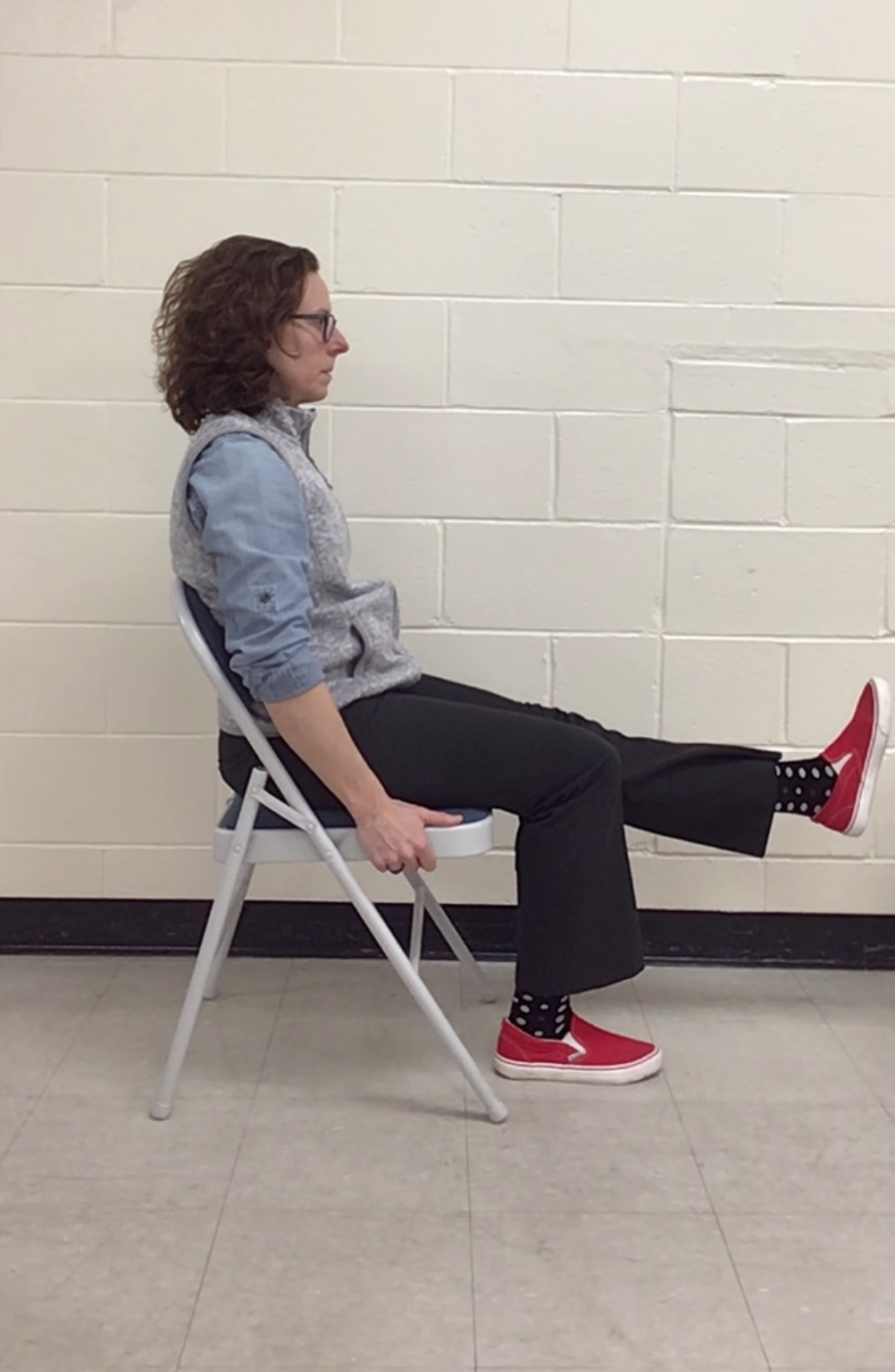  side  view  front  view |
| 1. **Seated hip abduction**  - Sit upright with back supported by the chair, knees bent, and feet flat on floor. - Keeping knee bent, lift one foot a few inches off the floor, move entire limb out to one side and tap foot on floor. Return to starting position - Repeat 10 repetitions on the right, then rest 10 seconds and perform 10 repetitions on the left.   Perform 3 sets on each side (Total = 30 on each side) | 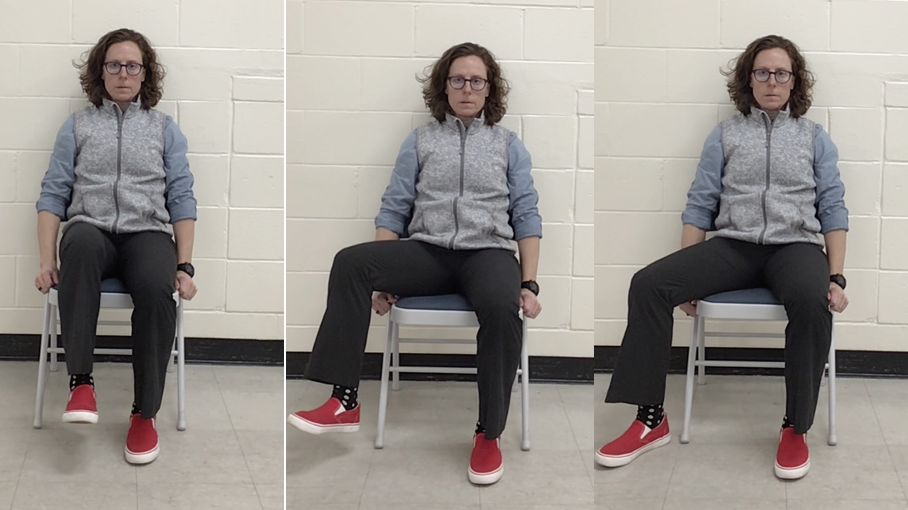  tap foot on floor, then return to start position  move leg to side  lift foot |
