# Supplementary material for: The effects of intrinsic foot muscle strengthening interventions for adults over age 65: a randomized controlled trial protocol
Source: Front Aging. 2025 Oct 15;6:1622232. doi: 10.3389/fragi.2025.1622232 (PMC12568628; doi:10.3389/fragi.2025.1622232)
Supplement: Supplementary file 4 [file Supplementaryfile2.docx]

**Foot Exercise Instructions**

For the next 4 months:

- Please do each of these **exercises** **1 time per day, 5 days per week**
- Record your performance in your Daily Exercise and Falls Journal

Your start date is ___________________ The end of your 4-months date is __________________

After the 4 months date, please continue the exercises 2 days per week until your end date.

Your study end date is _____________________.

We will see you in person 1 week and 4 weeks after you start this program. At those times we can answer any questions, and we will observe your technique with the exercises.

1 week date_________________ 4 weeks date __________________

We will also contact you by phone, text, or email (your preference) every 2 weeks throughout the study.

We will perform in-person measurement sessions at
week 8 date___________ week 16 date___________ & 1 year date _________________

Follow these instructions **5 days per week for 4 months (16 weeks)**. Do each foot separately.

| 1. **Doming or “shorten your foot”**  - Without clawing or bending your toes, press all toes into the ground and act as though you’re pulling your toes back toward your heel. - You should feel a pressure/tension in your arch.   Hold for 3 seconds  Repeat 10 repetitions  Perform 3 sets  (Total = 30 on each foot) | 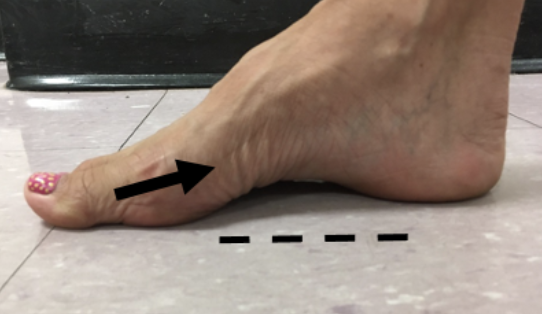 |
| --- | --- |
| 1. **Big toe lift**  - Gently press all toes into floor - Attempt to lift only your big toe while keeping small toes pressed into floor.   Repeat 10 repetitions  Perform 3 sets (Total = 30 on each foot) | 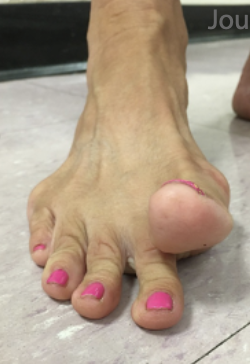 |
| 1. **Small toes lift**  - Gently press all toes into floor - Attempt to lift your 4 small toes while keeping the big toe pressed into the floor.   Repeat 10 repetitions  Perform 3 sets  (Total =30 on each foot) | 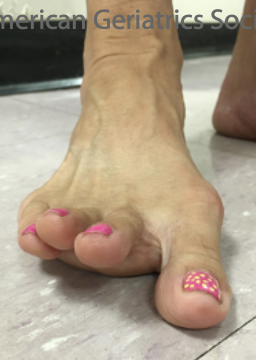 |
| 1. **Toe spread**  - Try to spread toes apart without lifting or bending them. - It’s ok if they don’t all visibly spread. Focus on moving just your big toe or just your little toe outward.   Repeat 10 repetitions Perform 3 sets (Total = 30 on each foot) | 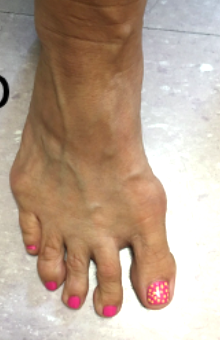 |
| 1. **Toe squeeze**  - Try to squeeze all toes together without lifting or bending them. - It may help to focus only on squeezing your big toe and second toe.   Repeat 10 repetitions  Perform 3 sets  (Total = 30 on each foot) | 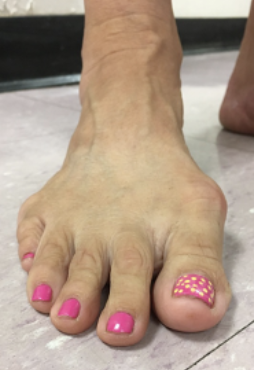 |
