# Supplementary material for: The effects of intrinsic foot muscle strengthening interventions for adults over age 65: a randomized controlled trial protocol
Source: Front Aging. 2025 Oct 15;6:1622232. doi: 10.3389/fragi.2025.1622232 (PMC12568628; doi:10.3389/fragi.2025.1622232)
Supplement: Supplementary file 5 [file Supplementaryfile4.docx]

**Semi-structured Interview for Foot Exercise Program**

1. What did you like about the exercises?

2. What did you not like about the exercises?

3. How much difficulty did you have with performing the exercises? (Use scale)

| 0 | 1 | 2 | 3 | 4 |
| --- | --- | --- | --- | --- |
| No difficulty | Mild difficulty | Moderate difficulty | Severe Difficulty | Unable to Perform |

4. *If participant responds 1-4 to #3*: Please describe what you found difficult about the exercises.

5. What things could be changed to make the exercises easier for people to perform?

6. What do you think would encourage people to perform these exercises consistently?

**Pedometer**

1. What did you like about the pedometer?

2. What did you not like about the pedometer?

3. How much difficulty did you have with using the pedometer? (Use scale)

| 0 | 1 | 2 | 3 | 4 |
| --- | --- | --- | --- | --- |
| No difficulty | Mild difficulty | Moderate difficulty | Severe Difficulty | Unable to Perform |

4. *If participant responds 1-4 to #3*: Please describe what you found difficult about the pedometer.

5. What things could be changed to make the pedometer easier for people to use?

6. What do you think would encourage people to use the pedometer consistently?

**Journal for Tracking Program and Falls**

1. How much difficulty did you have completing the journal? (Use scale)

| 0 | 1 | 2 | 3 | 4 |
| --- | --- | --- | --- | --- |
| No difficulty | Mild difficulty | Moderate difficulty | Severe Difficulty | Unable to Perform |

2. *If participant responds 1-4 to #3*: Please describe what you found difficult about completing the journal.

3. What could be changed to make the journal easier to use?

**Outcomes**

1. Pain:
   1. Did you have any low back or lower extremity pain when you started this study?
   2. Do you feel the foot exercises changed that pain? (Describe)
   3. Did the foot exercises cause any new pain?

2. Balance:

Do you feel the foot exercises changed your balance? (Describe)

3. Awareness of your feet:

Do you feel the foot exercises changed your awareness or sensation of your feet? (Describe)

**Student Experience**

1. What did you like about your experience working with the physical therapy students?

2. What did you not like about your experience working with the physical therapy students?

3. Would you change anything about your experience working with the physical therapy students?

**Other**

Is there anything else you would like to comment on or tell us about your experience with the study so far?
